# Sudden Cardiac Death in Childhood: Peaks in Teenagers

**DOI:** 10.1161/CIRCEP.124.013355

**Published:** 2025-02-10

**Authors:** Joseph D. Westaby, Mary N. Sheppard

**Affiliations:** Cardiac Risk in the Young (CRY) Centre for Cardiovascular Pathology, Cardiovascular Clinical Academic Group and Cardiology Research Section, Cardiovascular and Genomics Research Institute, St George’s University of London, United Kingdom. St George’s University Hospitals NHS Foundation Trust, London, United Kingdom.

**Keywords:** autopsy, cardiomyopathies, child, death, sudden, cardiac, pathology

Sudden cardiac death in children (SCDC) is a rare but devastating event estimated to occur at 0.7 to 10.1 per 100 000.^1,2^ Previous studies have highlighted sudden arrhythmic death syndrome (SADS) and cardiomyopathy as predominant causes.^1,2^ These causes are genetic and have major implications for surviving family. We aimed to assess the age, sex, and causes of SCDC from a specialist UK national cardiac pathology referral center.

This is a descriptive cohort study conducted in the Cardiac Risk in the Young cardiovascular pathology center based in St George’s University of London with institutional review board and ethical approval (10/H0724/38). Supporting data are available upon request. The center receives referrals from the United Kingdom where a cardiac cause of death is suspected or no cause is found at the original autopsy. We acknowledge that there may be a referral bias toward more complex cases. All referrals underwent full autopsy and toxicology to exclude noncardiac causes. SCDC was defined as death within 24 hours of being well in individuals aged >1 to 17 years. Information including symptoms was provided by referring coroners, family questionnaires, and general practitioners. Hearts were assessed according to guidelines as published previously.^3^ Categorical data are presented as number (%) and continuous data as mean±SD.

Between 2013 and 2022, there were 791 deaths of children with cardiovascular codes, and we received 415 (52%) cases of SCDC. There were 8108 referrals of sudden cardiac death between 1994 and 2023 and 624 (8%) were SCDC. Average age is 12±5 years with 413 boys and 211 girls (2:1). The Figure shows the age referral pattern by sex. SCDC peaks at the age of 1 year and between 13 and 17 years. SCDC in females starts to increase from 9 years of age, whereas SCDC in males starts to increase from 10 years of age. Death occurred associated with exertion in 128 (21%). Cardiac symptoms were reported in 71 (11%) with syncope/seizure/convulsion in 42 (7%), palpitations in 14 (2%), breathlessness in 11 (2%), and chest pain in 10 (2%). Abdominal pain was reported in 12 (2%).

**Figure.**
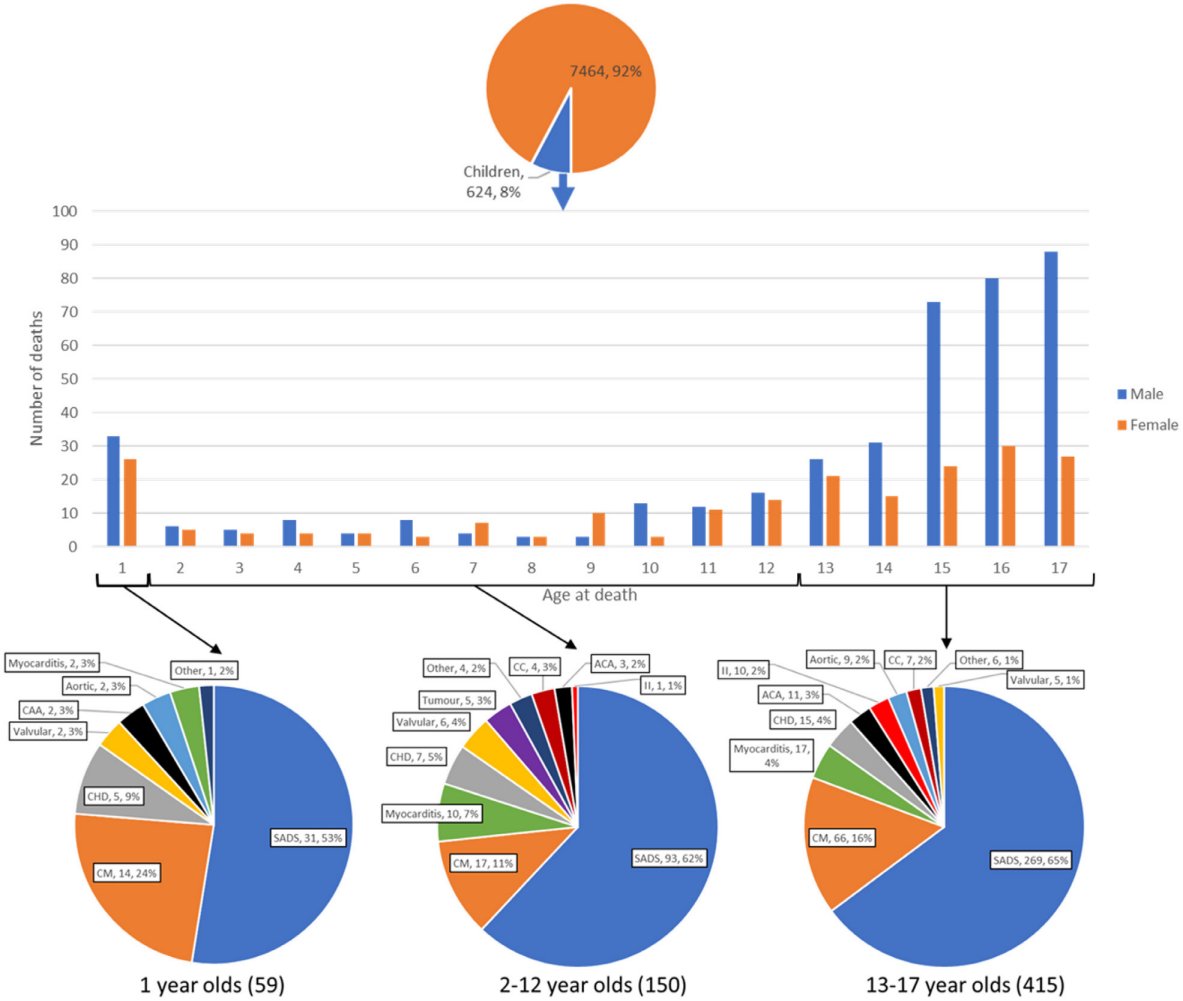
**Age, sex, and causes of sudden cardiac death in children (SCDC).** Of 7464 sudden cardiac deaths, 624 (8%) were in children. Sudden cardiac death in children peaks at the age of 1 year and between 13 and 17 years. Sudden arrhythmic death syndrome (SADS) increased as a proportion and congenital heart disease (CHD) decreased as a proportion of SCDCs with increasing age. Cardiomyopathy (CM) appears prominent at ≥1-year age group. Myocarditis made up a higher proportion of SCDCs between 2 and 12 years. Commotio cordis (CC) only appears in older children active in sport. ACA indicates anomalous coronary artery; and II, idiopathic infarction.

SADS (393, 63%), where the heart is morphologically normal, is most common in all age groups followed by cardiomyopathy (97, 15%), myocarditis (29, 4%), and congenital heart disease (27, 4%). Anomalous coronary artery (16, 2%), valve disease (13, 2%), commotio cordis (11, 2%), and idiopathic infarction (11, 2%) made up similar proportions. Rarer causes include cardiac tumor (5, 1%), Wolff-Parkinson-White syndrome (4, 1%), ischemic heart disease due to coronary artery atheroma (2, <1%), vasculitis (2, <1%), transplant vasculopathy (2, <1%), and hypertensive heart disease (1, <1%).

Cardiomyopathies include hypertrophic cardiomyopathy (25, 4%), arrhythmogenic cardiomyopathy (22, 4%), idiopathic fibrosis (14, 2%), idiopathic hypertrophy (12, 2%), dilated cardiomyopathy (10, 2%), and metabolic cardiomyopathy (5, 1%).

SADS increased as a proportion and congenital heart disease decreased as a proportion of SCDC with increasing age. Cardiomyopathy appears prominent at ≥1-year age group. Myocarditis made up a higher proportion of SCDC between 2 and 12 years. Commotio cordis only appears in older children active in sport.

In this study, SCDC peaks at the age of 1 year and between 13 and 17 years of age. SCDC is very rare between 2 and 8 years and increases dramatically in teenagers. This may suggest an influence of hormonal change raising the risk of SCDC especially in SADS and cardiomyopathy. During the pubertal growth phase, cardiac enlargement and increasing blood pressure place additional demand upon the heart, which may result in an increased risk of arrhythmia and SCDC.^4^ Alternatively, these deaths may be related to factors such as increased participation in strenuous sports or disease progression as in arrhythmogenic cardiomyopathy.^5^

There has been increasing interest in SCDC with link to heritable channelopathies and cardiomyopathies, risk stratification, genetic testing, and management. The nationwide Danish study reported 87 SCDCs, which agreed with main causes being SADS and cardiomyopathies. Our study reported less cardiac symptoms (11% versus 59%), but this is partially explained by the fact that they reported on all symptoms including stomach pain, back pain, and nausea.^1^ Stomach pain was their most common prodromal symptom; however, this was reported at a lower frequency in our cohort (2% versus 12%). We report a higher proportion of SCDC associated with exertion (21% versus 14%). They had more congenital heart disease in their cohort (4% versus 20%). In contrast to our study and another UK study,^2^ they found no hypertrophic cardiomyopathy.

A recent UK study of 151 SCDCs from 2 pediatric centers that had similar but contrasting findings found more congenital heart disease than cardiomyopathy, which may reflect specialist pediatric referral practice and including infants.^2^ Full autopsy and cardiac examination in all cases of SCDC is essential to make the correct diagnosis.

In this cohort study, a larger proportion of SCDCs were among teenagers raising a potential association with age. Most SCDCs are not associated with exertion (21% with exertion) or reported cardiac symptoms (11% with symptoms). In this cohort, SADS (63%) and cardiomyopathy (15%) accounted for a majority of SCDC, suggesting heritable conditions were the main cause of SCDC. Thus, families in which an inherited cardiac condition is identified in a child need screening to detect and treat these conditions to prevent further deaths. Based on the findings in this cohort, related children should be screened before 13 years of age.

## Article Information

### Sources of Funding

Cardiac Risk in the Young (CRY) funds the CRY cardiac pathology laboratory. Dr Westaby is supported by the National Institute for Health and Care Research. For the purpose of open access, the author has applied a Creative Commons Attribution (CC-BY) license to any author accepted manuscript version arising.

### Disclosures

None.
